# Intrinsic role of coagulase negative staphylococci *norA*-like efflux system in fluoroquinolones resistance

**DOI:** 10.3934/microbiol.2017.4.908

**Published:** 2017-11-14

**Authors:** Ligozzi Marco, Galia Liliana, Bertoncelli Anna, Mazzariol Annarita

**Affiliations:** Department of Diagnostics and Public Health, University of Verona, Verona, Italy

**Keywords:** *S. epidermidis*, *NorA*-like gene, efflux pumps, *S. haemolyticus*, coagulase negative staphylococci

## Abstract

NorA is a *Staphylococcus aureus* multidrug transporter that exports structurally distinct compounds including fluoroquinolones. In this study *norA-*like genes of *Staphylococcus* *epidermidis* (*norA*_SEP_) and *Staphylococcus haemolyticus* (*norA*_SHAE_) were identified and sequenced. The nucleotide identity of *norA*_SEP_ and *norA*_SHAE_ with *norA* was 75.3 and 74.1%, respectively, and the amino acid identity 87.7 and 86%, respectively. Inactivation of *norA*_SEP_ increased the ciprofloxacin susceptibility of *E. coli* DH5α carrying the pB SK 198 *norA*_SEP_ EZ cat *norA*_SEP_ plasmid.

## 1. Introduction

Efflux-mediated fluoroquinolone resistance has been described in Gram-positive species [1]. In *Staphylococcus* *aureus, Streptococcus pneumoniae*, viridans streptococci, enterococci, and *Bacillus subtilis* FQ exporting systems belong to the MSF family, the best characterized being NorA of *S. aureus* and Bmr/Blt of *B. subtilis*, responsible for resistance to FQ, basic dyes, puromycin, chloramphenicol, and tetraphenylphosphonium [2],[3].

The most information about the efflux-mediated mechanisms of FQR in staphylococci is available for *S. aureus.* The *norA* gene is expressed weakly in wild-type *S. aureus* cells, and *norA*-mediated resistance probably depends upon mutational upregulation of the gene expression, concomitant increase in production of the *norA* efflux pump [4],[5] and target site mutations.

Less is known about *Staphylococcus* *epidermidis* and other coagulase-negative staphylococci (CoNS). Target site mutations have been described [6]. Active efflux, as suggested by blocking by reserpine, contributes substantially to the resistance phenotype in some strains of CoNS [7],[8], and role of efflux overexpression of a mutation in an untraslated sequence before *norA*-like gene [9].

The present study was undertaken to investigate the role in intrinsic fluoroquinolones resistance of homologues of *norA* MFS-type efflux transporter in CoNS, namely *S. epidermidis* and *S. haemolyticus.* For this purpose, the *norA*-like gene was first sequenced, insert in a plasmid and then cloned in *E. coli* DH5α and finally the cloned gene was destroyed by transposon mutagenesis.

## 2. Materials and Method

The presence of *norA-*like sequences was investigated in CoNS strains from our collection (*S. epidermidis* 198, *Staphylococcus capitis* 92, *Staphylococcus chonii* 147, *Staphylococcus haemolyticus* 256) using PCR and degenerate oligonucleotide primers based on the highly-conserved motif of MSF-type efflux pumps (*norA* deg1: 5′-AATGTTTCAAAWGCAGAT-3′; *norA* deg2: 5′-KTTGCWGGWRCATTAGGT-3′, W = A, T; K= G, T; R = A, G). PCR was performed in 0.2 ml tubes in an MJ Research (BioRad, Hercules, CA). Standard PCR reactions were carried out in 50 µl with the following final concentrations: 50 mM KCl, l0 mM Tris-HCl (pH 9.0 at 25 °C), 0.1% Triton X-I00, 1.5 mM MgC1_2_, 100 µM each of dNTP, 0.5 µM of each primer, 0.5 U AmplTaqGold DNA polymerase (Applied Biosystems, Foster City, CA). Standard amounts of DNA were added: 30 ng genomic DNA or plasmid DNA. PCR cycling conditions were as follows: an initial denaturation at 94 °C for 5 min followed by 5 cycles of 94 °C for 30 sec, annealing at 37 °C and extension at 72 °C for 60 sec. This was followed by 30 cycles consisting of 94 °C for 30 sec, 50 °C for 30 sec and 72 °C for 60 sec and a final 5-min extension step at 72 °C.

Transposon mutagenesis, performed with EZ:TN transposon system (Epicentre, Biotechnologies, Madison, WI) was used to obtain a *norA*_SEP_ mutant in accordance with the manufacturer's instructions. The entire *norA-*like gene of *S. epidermidis* (*norA*_SEP_) was amplified using primers derived from the gene sequence (*norA*_SEP_ fw 5′-CATAACCACGCACTACTTTCT-3′; *norA*_SEP_ rev 5′-GACACAGAATTCGTCTTGAAC-3′) and cloned in the pBluescript SK plasmid (Stratagene, La Jolla, CA), resulting in plasmid pB SK 198 *norA*_SEP_ Transposon insertion into *norA*_SEP_ was done by incubating the plasmid containing *norA*_SEP_ with an equal molar amount of the EZ:TN <CAT> transposon, encoding chloramphenicol resistance, and EZ:TN transposase for 2 h at 37 °C according to the manufacturer's instructions. Following transformation of chemically competent *E. coli* DH5α cells (Stratagene, La Jolla, CA) with *in-vitro* insertion reaction, clones were selected by growth on 10 µg/ml chloramphenicol agar plates. Chloramphenicol-resistant clones were submitted to PCR analysis with *norA*_SEP_ primers.

The MIC of ciprofloxacin for both *E. coli* DH5α and *S. epidermidis* 198 were determined in triplicate with E-test strips according to the EUCAST guidelines [10].

SDS-polyacrylamide gel electrophoresis of NorA of *E coli* DH5α wild-type and harboring the recombinant plasmid. pB SK 198 *norA*_SEP_ was done. *E. coli* strains were grown in LB broth (5 ml) with ampicillin (100 µg/ml) at 37 °C with shaking (300 rpm). At the absorbance of 600 nm, cells were harvested and resuspended in a 0.1 volume of loading buffer and incubated for 2 min at 100 °C.

## 3. Results

An amplification product of 190 bp from total DNA both of S. *aureus* SA 1199 (kindly provided by G. W. Kaatz), and of different species of the CoNS were obtained.

S. *epidermidis* 198 was selected for subsequent cloning experiments. The 190 bp PCR product was sequenced by the *Taq* dye-deoxy terminator method with a 377 DNA Sequencing System (Applied Biosystems, Foster City, CA). Sequence analysis and alignments were done using the Genebase version 1 computer software (Applied Maths, Kortrijk, Belgium) and revealed a high degree of homology with the corresponding sequence of *S. aureus norA.*

To perform the complete sequence of the *norA-*like gene an inverse PCR approach [11] starting from the 190 bp sequence found in the *S. epidermidis* 198 chromosome was followed. The *norA*-like gene was found to be located in a 1.7-kb fragment whose nucleotide sequence showed one open reading frame (nucleotides 568 to 1728) long enough to encode a polypeptide of 387 amino acids (accession number AJ621598). Putative promoter sequences were found at nucleotides 450 to 455 (TACAAT) and nucleotides 426 to 431 (TTGTCA), which well match the consensus sequences (TATAAT and TTGACA) for the −10 and −35 regions of *E. coli* promoters. An inverted repeat, which might act as a transcription terminator, was found at nucleotides 1797 to 1834. The gene was 1161 bp nucleotides in length and consisted of 387 amino acids. The sequence revealed a nucleotide identity of 75.3% with *norA* of *S*. *aureus* 1199. The complete *norA* gene of *S*. *epidermidis* 198 was designated as *norA*_SEP_. Using a similar strategy, the sequence of the *norA-*like gene of *S. haemolyticus* 256 (*norA*_SHAE_) was also performed (accession number AJ621601).

**Figure 1.**
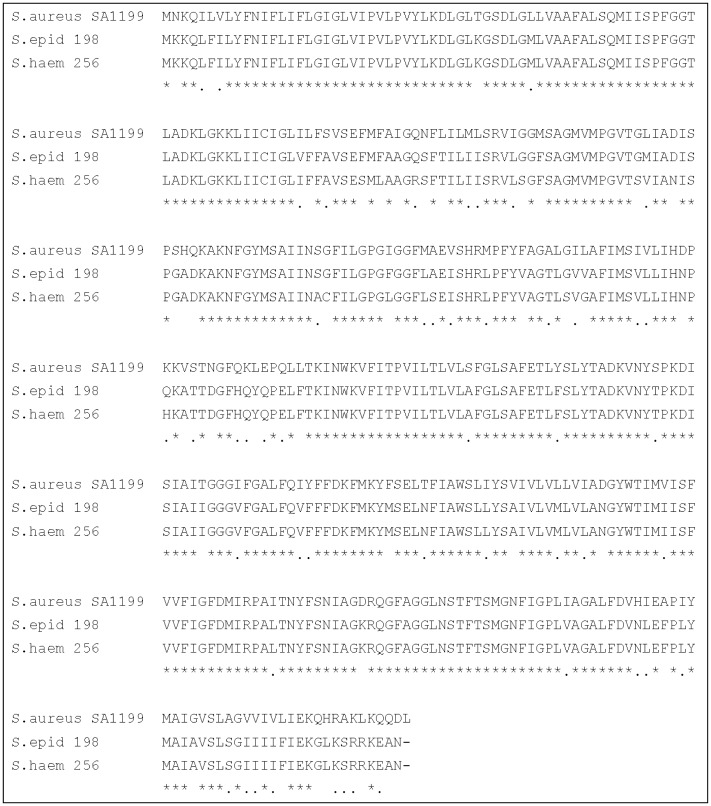
The deduced amino acid sequence alignment of *S. aureus* NorA, amino acid sequence, *S. epidermidis* 198 NorA and *S. haemolyticus* 256 NorA alignments. Asterisks and dots indicate residues that are identical and similar to the three amino-acids sequences respectively.

Figure 1 shows the alignments between the deduced amino-acid sequence of *S. aureus* NorA protein and that of NorA_SEP_ and NorA_SHAE_. The nucleotide identity of *norA*_SEP_ and *norA*_SHAE_ with *norA* was 75.3 and 74.1% and the amino-acid identity 87.7 and 86%, respectively. These results indicate a high degree of homology between the *norA* genes of CoNS and the *norA* gene of *S. aureus.*

The insertional mutagenesis approach to inactivate the *norA* gene was used to determine the physiological function of the protein encoded by *norA*_SEP_.

Several mutants of *S. epidermidis* were obtained by *in-vitro* transposition technics [12]. The entire *norA*_SEP_ gene was amplified using primers derived from the gene sequence and cloned in the pBluescript SK plasmid (Stratagene, La Jolla, CA), resulting in plasmid pB SK 198 *norA*_SEP_. Transposon insertion into *norA*_SEP_ was done with the EZ:TN <CAT> transposon and clones in chemically competent *E. coli* DH5α cells (Stratagene, La Jolla, CA) with *in-vitro* insertion reaction were selected by growth on 10 µg/ml chloramphenicol agar plates. Chloramphenycol-resistant clones were submitted to PCR analysis with *norA*_SEP_ primers. Insertion of the transposon in *norA*_SEP_ increased the amplicon length from 1.6 kb (*norA*_SEP_ gene without the transposon insertion) to 2.4 kb. Several clones were obtained which gave amplicons of the expected length. From one of these clones (*E. coli* DH5α 198 *norA*_SEP_) the recombinant plasmid containing the *norA*_SEP_::*cat* fragment (pB SK 198 *norA*_SEP_ EZ cat) was purified and sequenced.

In Table 1 are reported the results of MICs of some fluoroquinolones that are NorA efflux substrate as ciprofloxacin, levofloxacin and ofloxacin. MICs are measured also for substrate as the moxifloxacin that effect NorB but NorA efflux pumps. All antibiotics were tested alone and in presence of carbonyl *m*-chlorophenylhydrazone (CCCp) an efflux pumps inhibitor. *E. coli* DH5α carrying the plasmid pB SK 198 *norA*_SEP_ had a ciprofloxacin MIC of 0.25 µg/ml, eight times higher than the MIC of *E. coli* DH5α carrying the plasmid pB SK 198 *norA*_SEP_ EZ cat *norA*_SEP_ (MIC = 0.032 µg/ml) and *E. coli* DH5α without plasmid. Similar effect is register for levofloxacin and ofloxacin. There are no effects indeed in the MICs of moxifloxacin as well the tetracycline, since they are not substrates of NorA pump.

**Figure 2.**
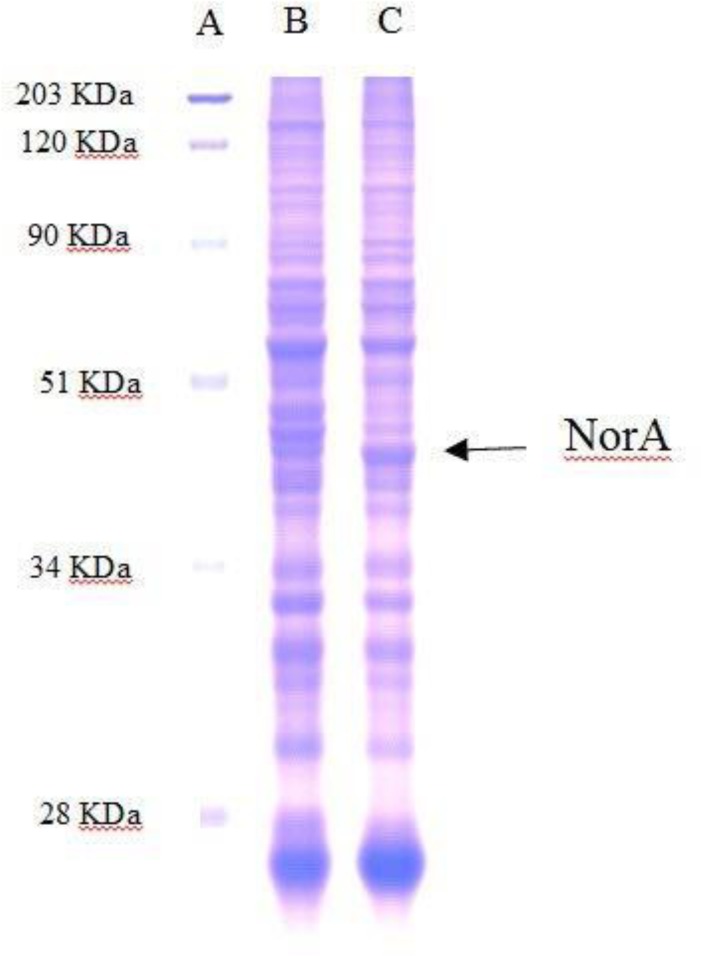
SDS-PAGE and NorA expression analysis in *E coli* DH5α. Line A: Pre-stained Molecular Weight (Biorad, Milan Italy); line B: *E. coli* DH5α recipient strain; line C: *E. coli* DH5α pB SK 198 *norA*_SEP_.

In order to confirm expression of the efflux pump protein we performed a SDS-polyacrylamide gel electrophoresis (PAGE) and NorA expression analysis in *E coli* DH5α wild-type and harboring the recombinant plasmid pB SK 198 *norA*_SEP_. In Figure 2 we showed the presence of aroung 42 KDa protein in the strains harboring the recombinant plasmid only.

**Table 1.** MICs value of *E coli* DH5α and its trans-conjugants for fluoroquinolones and tetracycline, in absence and presence of CCCp.

Strains	MIC (mg/L)									
	Ciprofloxacin		Levofloxacin		Ofloxacin		Moxifloxacin		Tetracycline	
	−CCCp	+CCCp^a^	−CCCp	+CCCp^a^	−CCCp	+CCCp^a^	−CCCp	+CCCp^a^	−CCCp	++CCCp^a^
*E. coli* DH5α	0.032	0.032	0.015	0.015	0.015	0.015	0.0075	0.0075	0.5	0.5
*E. coli* DH5α pB SK 198 *norA*_SEP_	0.25	0.015	0.12	0.015	0.06	0.015	0.015	0.015	1	1
*E. coli* DH5α pB SK 198 *norA*_SEP_ EZ cat	0.032	0.032	0.015	0.015	0.015	0.015	0.015	0.015	0.5	0.5

^a^ Carbonyl *m*-chlorophenylhydrazone (CCCp) was added with concentration of 1 µg/ml.

## 4. Conclusion

Our results demonstrated that *norA-*like genes play an important role in the intrinsic FQR in CoNS. The resistance level to FQ due to NorA efflux pumps is not elevated. Like in other species such as *S. aureus*, *S. pneumoniae* the overexpression of efflux pumps combined with other mechanisms may contribute to increase the resistance at high level.
